# Steady State Ocean Response to Wind Forcing in Extratropical Frontal Regions

**DOI:** 10.1038/srep28842

**Published:** 2016-06-29

**Authors:** Meghan F. Cronin, Tomoki Tozuka

**Affiliations:** 1 NOAA Pacific Marine Environmental Laboratory, Seattle, WA, USA; 2 Department of Earth and Planetary Science, Graduate School of Science, The University of Tokyo, Tokyo, Japan

## Abstract

In regions of strong sea surface temperature (SST) gradients, the surface “geostrophic” currents have a vertical shear aligned with the surface density front defined by the temperature. This surface geostrophic (“thermal wind”) shear can balance a portion of the surface wind stress, altering the classic Ekman response to wind forcing. Here we show that these frontal effects cannot be ignored in the Tropics or in strong frontal regions in the extratropics, such as found in coastal regions and in western boundary currents of all basins. Frontal effects also dominate the classic Ekman response in the regions of both hemispheres where Trade winds change to westerlies. Implications for vertical motion and global heat transport are discussed.

Wind forcing is fundamental to the ocean circulation. Our understanding of it, developed in the early twentieth century^1^, assumes that the wind is acting on a homogeneous ocean (i.e., without fronts), of constant viscosity: Due to Coriolis turning, in the Northern Hemisphere, wind-induced steady flow spirals to the right of the wind stress, resulting in a net wind-forced “Ekman” transport that is 90 degrees to the right of the wind stress and is proportional to the wind stress and inversely proportional to the Coriolis parameter. In the Southern Hemisphere, the Coriolis parameter is negative and the spiraling and net Ekman transport is to the left of the wind stress. Although both the homogeneous and constant viscosity assumptions are met in only limited regions of the global ocean, the Ekman theory is applied almost universally. Indeed, many accountings of the global ocean heat balance assume that surface temperature fronts are uniform with depth in the upper ocean and are advected by the classic Ekman transport, even though the classic Ekman transport was derived for regions where no horizontal density gradients exist^2^,^3^.

Using a suite of meteorological sensors and a set of five current meters with temperature sensors mounted on the upper 25 m of a surface mooring in the Pacific at 2°N, 140°W, Cronin and Kessler^4^ showed that, in fact, the wind-driven Ekman response was highly sensitive to the presence of the equatorial cold tongue’s front. As a result, the near surface currents did not display classic Ekman wind response characteristics. Over the 5-month period, the mean surface currents at 2°N, 140°W tended to spiral to the left of the winds with depth, even though this was in the Northern Hemisphere. The mystery was resolved with the realization that a large vertical shear in the geostrophic currents would be aligned with the front in the SST. In the atmosphere, this geostrophic shear is referred to as thermal wind shear, and the term is commonly used for oceanic geostrophic shear as well.

At the surface of the ocean, wind stress (**τ**_**0**_) is required to balance the surface vertical shear (∂***u***/∂*z*), with the proportionality constant equal to the turbulent eddy viscosity (*v*) multiplied by the background density *ρ*_*0*_: ***τ**_**0**_* = *ρ*_0_*v* ∂***u/***∂*z*. In the classic Ekman theory, the fluid is homogenous and the wind stress induces an ageostrophic shear that accounts for the total surface shear. However, in frontal regions, the shear that balances the wind stress will have a geostrophic component and only a portion of the wind stress induces the ageostrophic surface shear and Ekman spiral: the portion of the wind stress that is out of balance with the surface geostrophic shear 
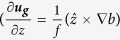
, where *f* is the Coriolis parameter and *b* is the sea surface buoyancy, which is a function of temperature and salinity – see Methods). We call this the “effective wind stress”:


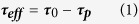


where 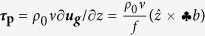 is the stress associated with the pressure gradient-induced geostrophic shear, referred to hereinafter as the geostrophic shear stress. As shown by Cronin and Kessler^4^, the ageostrophic shear, induced by the effective wind stress, added to the geostrophic shear, produced currents that spiraled to the left of the wind stress in agreement with the observations. Note that equation (1) can be rearranged to highlight the importance of the frontal effects relative to the classic Ekman dynamics:


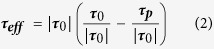


The first term in brackets on the right-hand side of equation (2) is the unit vector in the direction of the wind stress. The second term is the ratio of the geostrophic shear stress magnitude relative to the wind stress magnitude 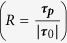 in the direction parallel to the front. If this ratio *R* is large relative to 1, then frontal Ekman dynamics dominate.

Because the Coriolis parameter approaches zero near the equator, the geostrophic shear stress (which scales as 1/*f*) and frontal effects indicated by *R* are very large in the tropics. For this reason, the classic Ekman theory is generally not applied to the equator and frontal effects on the Ekman response are commonly ignored outside of the tropics. As we show, however, homogeneity is a poor assumption in many key parts of the global ocean. In this study we investigate how important these frontal effects may be on the Ekman response in extratropical regions. For this analysis, data from the high-resolution Japanese Ocean general circulation model For the Earth Simulator (OFES) are used (see Methods for a brief description of OFES). Figure 1 shows the mean wind stress, the stress associated with the surface geostrophic shear, and the ratio of their magnitudes, *R*. The prevailing winds are predominately zonal over most of the open ocean and their magnitude has a meridionally banded structure associated with the westerlies at mid-latitudes and easterly trade winds at low latitudes. Critically, at the transition latitudes between westerly and easterly prevailing surface winds, the magnitude of the wind stress is very weak and in all basins, frontal effects dominate along these transition zone swaths (Fig. 1d), even though the surface density gradients are relatively weak.

As expected, the surface geostrophic shear stress is largest in the tropics (Fig. 1c), and thus frontal effects on the Ekman flow are largest near the equator (Fig. 1d). However, the geostrophic shear stress is also large in extratropical frontal regions (Fig. 1a,c), such as in coastal regions of most basins, and the frontal regions between the subtropical gyres and subpolar gyres of all basins (Fig. 1a). These extratropical fronts are particularly strong in the Southern Ocean and in western boundary current extension regions, such as the Kuroshio Extension in the North Pacific, the Gulf Stream of the North Atlantic, Agulhas Return Current of the South Indian Ocean, and the Brazil-Malvinas Current of the South Atlantic. In these extratropical frontal regions, as well as in the transition zones for wind, frontal effects on the Ekman ocean response can be order one (Fig. 1d).

In Fig. 2, we zoom into the western North Pacific region to see the multiscale structure associated with the frontal effects. In the wind transition zone where winds are very weak, the frontal Ekman effects are basin-scale, extending in a narrow strip from the western boundary region to mid-basin. The solid line in Fig. 2 indicates the location of the mean zero wind stress. Curiously, the mean surface shear is not zero here, even though the boundary condition at every model time step will set the surface shear to be zero if the surface wind stress is zero. Because viscosity and wind stress both co-vary on seasonal time scales, the zero surface shear is located somewhat to the north of the mean wind stress zero line. In the Kuroshio Extension region, the frontal effects are large scale, with evidence of synoptic scales associated with standing meanders in the mean current. In coastal regions, we see narrow bands where strong filament fronts dominate.

Zooming in again, in Fig. 3 we consider the relation between the wind stress, effective wind stress, and the surface shears at locations within the wind transition region and the Kuroshio frontal region for months where the climatological fronts are strongest. In particular, the surface currents relative to those at 24 m (which are proportional to shear) are decomposed into geostrophic and ageostrophic components. In both regions, the surface geostrophic shear stress is larger than the wind stress. Consequently, the effective wind stress is dramatically different from the wind stress. As shown in Fig. 3 in both locations, the ageostrophic surface current relative to 24 m is roughly aligned with or slightly to the right of the effective wind stress, consistent with the ageostrophic current being induced by the effective wind stress. In the wind transition zone where winds are very weak, the geostrophic shear stress associated with the surface meridional density gradient will not be balanced by a wind stress and will thus induce an ageostrophic secondary circulation. This secondary circulation is consistent with the response of an idealized 2-dimensional modeled frontal zone with zero wind stress applied^5^. The extended swath of high *R* values associated with the transition zone shown in Figs 2 and 3 suggests that this secondary circulation extends across much of the basin. Likewise in the Kuroshio frontal region, the very strong northeastward geostrophic shear that is out of balance with the southeastward wind stress induces an ageostrophic shear that is large and counter to the surface geostrophic shear. The resulting vertical velocity along a meridional transect through the study site is shown in Fig. 4.

In classical Ekman theory, vertical motion (“Ekman pumping”) is directly related to the wind stress curl. As shown in the methods section, in frontal regions, Ekman pumping is instead related to the curl of the effective wind stress: Ageostrophic velocity is induced to the right (left) of the effective wind stress in the Northern (Southern) Hemisphere. Thus a curl in the effective wind stress will lead to a horizontal convergence/divergence of ageostrophic flow and, because the water is incompressible, vertical velocity. While the wind stress curl is broad, the effective wind stress is strongly influenced by the presence of the front and thus its curl also has frontal-scale variability. As shown in Fig. 4, the ocean indeed has a narrow band of upwelling on the warm side of the front and downwelling on the cold side. While the Ekman pumping inferred from the curl of the effective wind stress is significantly better than the classic Ekman pumping at capturing the order one features of the actual vertical velocity at the base of the mixed layer, there are still discrepancies. These may be due to the linear physics assumed here, and the simplifying assumptions that the front and eddy viscosity are vertically uniform. In contrast to the classic Ekman pumping velocity, the vertical motion is large at the base of the surface mixed layer and tends to deform the mixed layer upward on the warm side and downward on the cool side, acting to tilt the front into a vertical stratification. Intensified Ekman upwelling in frontal regions can result in an increase in nutrients available at the surface, as seen in chlorophyll images from space^6^. In addition, as shown by Tozuka and Cronin^7^, the resulting variations in mixed layer depth may also affect frontogenesis processes.

The concept that geostrophic shear associated with fronts can play a role in the Ekman response to wind forcing dates back to the early work of Garret and Loder^8^. The secondary circulations that can arise in frontal regions were explored in a semi-geostrophic model by Thompson^5^, an ocean general circulation model by Perez *et al*.^9^, and a high-resolution ocean model by McWilliams *et al*.^10^. In all cases, the Ekman flow tends to adjust to the front, its sheared flow distorting and tilting the front, allowing the Ekman vertical velocity to flow under and over the distorted front. The front is not a passive feature that can be advected by the classic Ekman transport. Indeed, based upon these arguments, the actual Ekman heat transport will likely be weaker than that estimated using the classical Ekman transport.

The classic Ekman theory has been used for over 100 years and therefore it is perhaps remarkable that it is not representative of the wind response in frontal regions. Fronts are ubiquitous in the world’s oceans^11^. It is important, though, to realize that the second major assumption of the classic Ekman theory, that the eddy viscosity is uniform with depth, is really only valid in the surface mixed layer. Below the mixed layer, eddy viscosity rapidly decays and the interior can be considered, to good approximation, inviscid. Thus, if the wind response is integrated to a deeper level where the eddy viscosity approaches zero, then the Ekman transport will revert back to the classic Ekman transport^4^,^12^. However over this depth the front is likely to differ from its value at the surface. For the purposes of global heat budgets, it may not be appropriate to assume that this is the transport that advects surface temperature gradients.

The role played by the eddy viscosity in shaping the Ekman response is difficult to overemphasize. As discussed by Wenegrat and McPhaden^12^, *R* is sensitive to the scaling assumptions for eddy viscosity. If, for example, eddy viscosity is such that the Ekman depth scales as u^*^/*f* (where 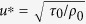), then *R* will be independent of wind stress and depend solely upon the density gradient and latitude. We find, however, that in the transition regions between the Trade winds and westerly winds, frontal Ekman dynamics can dominate, even though the surface density gradients there are not sharp. Indeed, our study shows that frontal Ekman dynamics are important throughout most of the tropics, in the transition zones between Trade wind and westerly winds, and in the frontal regions of western boundary currents and coastal regions. While previous investigations of these dynamics have typically focused on submesoscale eddies and filaments^10^, our study, which is based upon the high resolution OFES model, shows that these frontal Ekman dynamics can have basin scale features even in the extratropics.

This secondary circulation induced by the turbulent geostrophic shear should be distinguished from the secondary circulations associated with the effect of sea surface velocity on wind stress [e.g., refs 13 and 14]. Both are related to fronts. However in one case, the front affects the wind stress, which then affects the ocean response. In the other case, discussed here, the ocean surface shear adjusts to the wind stress. If the wind stress has been properly estimated to account for the difference between the air and water surface speeds, then both effects will be present. As models’ resolution becomes higher^15^ and observing techniques become more sophisticated, the distribution of heat and salt in the oceans becomes more inhomogeneous and fronts more ubiquitous. It is time that we put aside the assumptions of homogeneity.

## Methods

### Steady state, linear response to wind forcing (Ekman equations)

The basic equations considered are the horizontal momentum equation with the assumptions of steady state, linear motion in a viscous ocean subject to a wind stress (***τ***_***0***_):


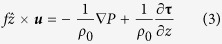


where *f* is the Coriolis parameter, 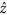 is the unit vector in the vertical direction,***u*** is the horizontal velocity vector, *ρ* and *ρ*_*0*_ are the actual and background water densities, *P* is pressure, and ***τ***is stress; and the vertical momentum equation, which under the assumption that the fluid is hydrostatic, simplifies to:


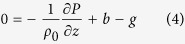


where *g* is gravity and *b* = *g*(*ρ*_*0*_ − *ρ*)/*ρ*_*0*_ is buoyancy. Equation (3) can be decomposed into a geostrophic steady-state flow (***u***_***g***_) that balances the pressure gradient and an ageostrophic “Ekman” flow (***u***_***a***_) that balances the stress divergence. This clean separation, however, breaks down in frontal regions when one considers the boundary condition that wind stress is proportional to the vertical shear, 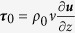 (where ν is the eddy viscosity), which rewritten in terms of the ageostrophic shear and combining equations (3) and (4) to write the geostrophic shear in terms of a buoyancy gradient, becomes:


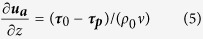


where 
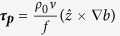
 is the geostrophic shear stress associated with sea surface density front. The “Ekman transport” (***U***_***a***_) can then be computed by vertically integrating the stress divergence term to a depth where the stress is zero:


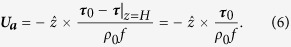


In classic Ekman conditions (homogeneous fluid of constant viscosity), *H* is the depth at which the ageostrophic shear spirals to an infinitesimally small value. Consequently, the stress at that level is negligibly small and the Ekman wind-driven transport is 90 degrees to the right of the wind in the Northern Hemisphere and 90 degrees to the left in the Southern Hemisphere (where *f* is negative). Most importantly, the wind-driven transport is determined entirely by the wind stress and latitude. In frontal regions, however, the depth at which the ageostrophic shear is negligible may not be a level of no stress. For example, if, instead of integrating to a level of no stress, one integrates to the base of a deep mixed layer in which both the front and eddy viscosity are uniform with depth, the stress at the depth *H* is then approximately ***τ***_***p***_: 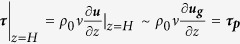. Thus, under these frontal conditions with uniform eddy viscosity, the Ekman transport (equation (6)) is related to the effective wind stress, rather than the total wind stress. If, however, viscosity is assumed to decrease with depth, then **τ**_p_ can become zero at *H*, even in regions of vertically uniform fronts. Therefore, even in frontal regions, if one considers the wind-generated transport down to the level where the flow is inviscid, the Ekman transport reverts to the classic Ekman value and is dependent only upon the surface wind stress.

Similarly, “Ekman pumping” (the vertical velocity associated with the wind forcing) is derived by computing the divergence in the ageostrophic velocity, and integrating from the surface to depth *H*:


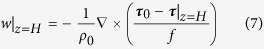


In the case that *H* is the base of the mixed layer with uniform eddy viscosity and front, 
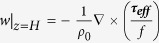
. While in the case that *H* is the depth at which the fluid becomes inviscid, equation (7) becomes the classic Ekman pumping equation: 
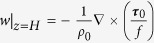
.

### OFES model

The Ocean model For the Earth Simulator (OFES)^16^ is based on the third version of the Modular Ocean Model (MOM3)^17^. Its horizontal resolution of 0.1° × 0.1° is sufficient to resolve surface frontal structures in the world ocean, and 54 vertical levels with 5 m resolution near the surface allows the model to simulate the upper ocean response to wind forcing relatively well. After a spin-up, the OFES is forced by the daily mean data from the National Centers for Environmental Prediction/National Center for Atmospheric Research (NCEP/NCAR) reanalysis data^18^ from 1950 to the present. We use model outputs from the 2000–2009 period to construct monthly mean climatologies. The model has relatively good skill in reproducing the frontal structures in the Kuroshio Extension region^19^. Eddy viscosity and diffusivity are calculated using the KPP model^20^. However since eddy viscosity was not saved during its integration, we have estimated surface eddy viscosity from *ν* = ***τ***_0_/(*ρ*_0_ *∂**u***/*∂*z), where we used the NCEP/NCAR reanalysis data for the surface wind stress and simulated oceanic currents at the first two vertical levels located at 2.5 m and 7.6 m depth to calculate the surface shear.

## Additional Information

**How to cite this article**: Cronin, M. F. and Tozuka, T. Steady State Ocean Response to Wind Forcing in Extratropical Frontal Regions. *Sci. Rep.* **6**, 28842; doi: 10.1038/srep28842 (2016).

## Figures and Tables

**Figure 1:**
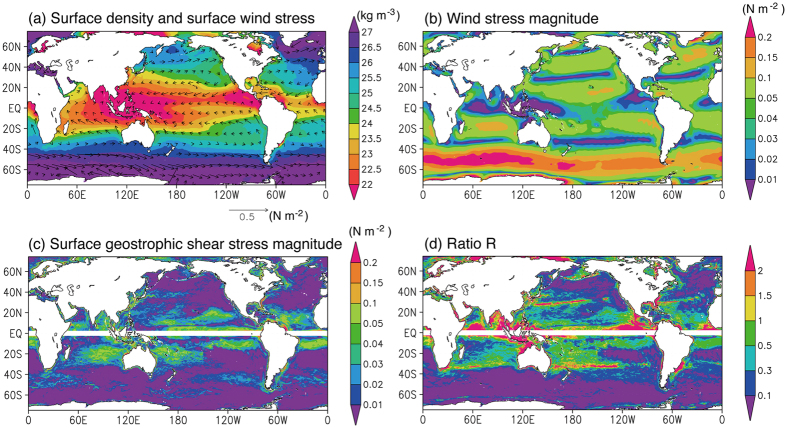
Global maps of the mean climatological (a) sea surface density and surface wind stress (*τ*_0_), (b) wind stress magnitude (|*τ*_0_|), (c) surface geostrophic shear stress magnitude (|*τ*_*p*_|), (d) and their ratio, *R* = |*τ*_*p*_|/|*τ*_0_|. All fields are from the OFES model. All plots were generated with GrADS v.1.9b4 (http://cola.gmu.edu/grads/grads.php).

**Figure 2:**
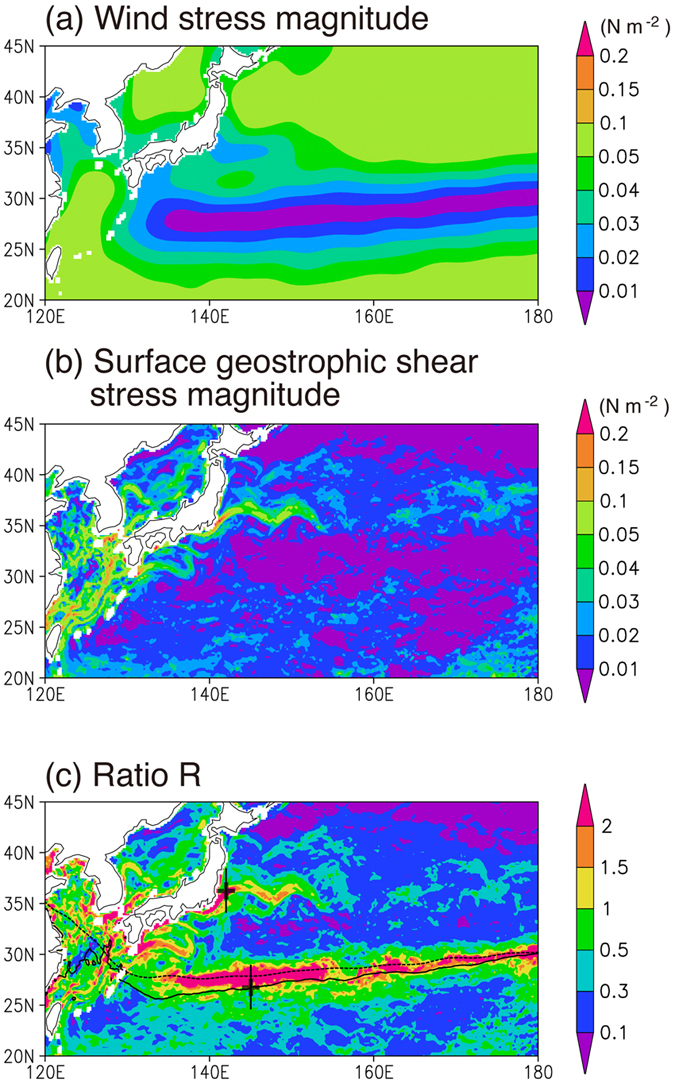
Climatological mean values in the Kuroshio Extension region of the northwest Pacific for (a) wind stress magnitude, (b) surface geostrophic shear stress magnitude, and (c) their ratio, *R*. The solid line in (**c**) shows the location of the mean zero wind stress. The dashed line indicates the mean zero surface shear. The study sites for Figs 3 and 4 are also shown in (**c**). All plots were generated with GrADS v.1.9b4 (http://cola.gmu.edu/grads/grads.php).

**Figure 3:**
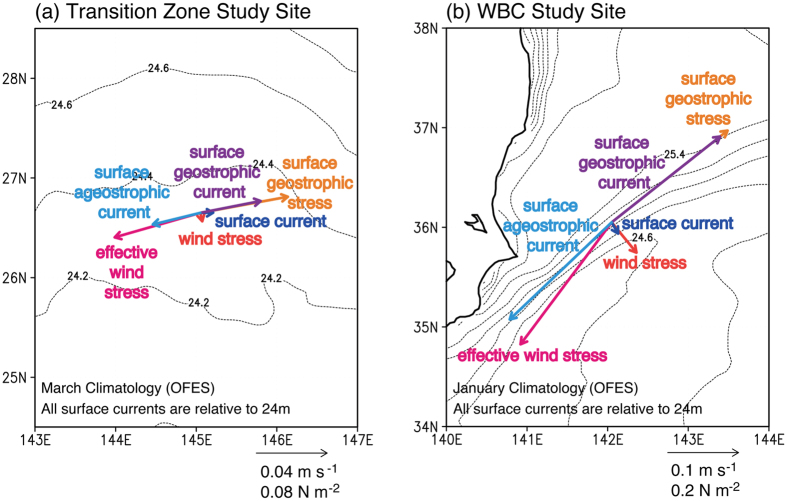
Surface stress (red) decomposed into surface geostrophic shear stress (orange) and effective wind stress (magenta) components and surface current relative to 24 m (blue) decomposed into geostrophic (purple) and ageostrophic (light blue) components for (a) a site in the transition zone at 145°E, 26.6°N, and (b) a site in the Western Boundary Current at 142°E, 36°N. Vectors in (**a**) are computed from March climatology values of OFES. Vectors in (**b**) are computed from January climatology values of OFES. All plots were generated with GrADS v.1.9b4 (http://cola.gmu.edu/grads/grads.php).

**Figure 4:**
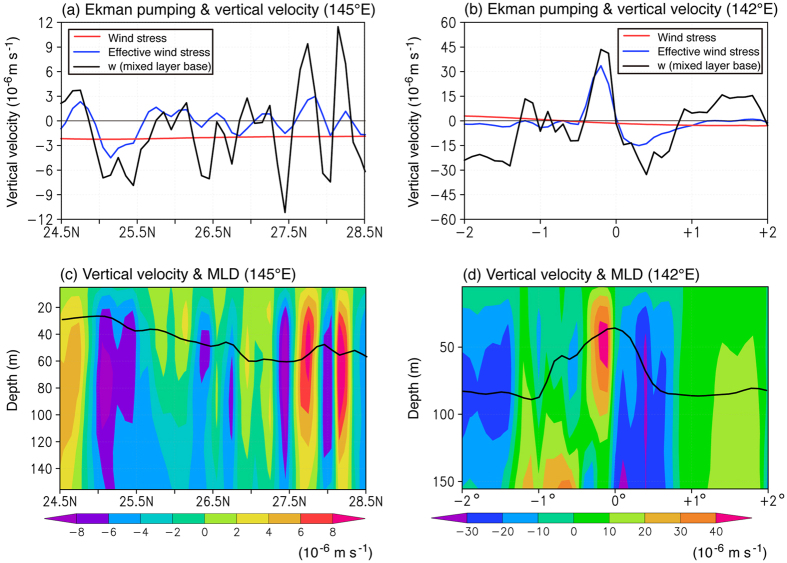
Vertical velocity along meridional sections shown in Fig. 2c. (**a**) The classic Ekman pumping velocity at the depth of no stress is shown in red and the vertical velocity computed using the effective wind stress curl (see Methods) is shown in blue for the transition region study site. The OFES model’s vertical velocity at the base of the mixed layer, which is defined by the depth at which the density becomes larger by 0.03 kg m^−3^ compared to the surface density, is shown in black. (**c**) The vertical velocity profile in the upper 150 m from the OFES model. The depth of the mixed layer base is shown as a black line. (**b,d**) Same as (**a**,**c**) but for the western boundary current study site. Units of vertical velocity are 10^−6^ m/s. All plots were generated with GrADS v.1.9b4 (http://cola.gmu.edu/grads/grads.php).
