# Erratum: Steady State Ocean Response to Wind Forcing in Extratropical Frontal Regions

**DOI:** 10.1038/srep30996

**Published:** 2016-08-01

**Authors:** Meghan F. Cronin, Tomoki Tozuka

Scientific Reports 6: Article number: 2884210.1038/srep28842; published online: 06 29 2016; updated: 08 01 2016

In the HTML version of this Article, there is a typographical error in the Introduction section,

“We call this the “effective wind stress”:


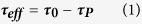


where 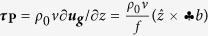 is the stress associated with the pressure gradient-induced geostrophic shear, referred to hereinafter as the geostrophic shear stress.”

should read:

“We call this the “effective wind stress”:


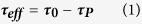


where 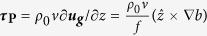 is the stress associated with the pressure gradient-induced geostrophic shear, referred to hereinafter as the geostrophic shear stress.”

